# Association of nasopharyngeal viruses and pathogenic bacteria in children and their parents with and without HIV

**DOI:** 10.1186/s41479-021-00088-5

**Published:** 2021-05-05

**Authors:** Tila Khan, Ranjan Saurav Das, Amrita Chaudhary, Jyotirmoy Chatterjee, Sangeeta Das Bhattacharya

**Affiliations:** School of Medical Science & Technology, Indian Institute of Technology Kharagpur, Kharagpur, West Bengal 721302 India

**Keywords:** HIV-infected children, Respiratory virus, Bacteria, Pneumococcus, Virus-bacteria interaction, Carriage density, Rhinovirus, Adenovirus

## Abstract

**Background:**

Bacteria and respiratory viruses co-occur in the nasopharynx, and their interactions may impact pathogenesis of invasive disease. Associations of viruses and bacteria in the nasopharynx may be affected by HIV.

**Methods:**

We conducted a nested case-control study from a larger cohort study of banked nasopharyngeal swabs from families with and without HIV in West Bengal India, to look at the association of viruses and bacteria in the nasopharynx of parents and children when they are asymptomatic. Quantitative polymerase chain reaction for 4 bacteria and 21 respiratory viruses was run on 92 random nasopharyngeal swabs from children--49 from children living with HIV (CLH) and 43 from HIV uninfected children (HUC)-- and 77 swabs from their parents (44 parents of CLH and 33 parents of HUC).

**Results:**

Bacteria was found in 67% of children, viruses in 45%, and both in 27% of child samples. *Staphylococcus aureus* (53%) was the most common bacteria, followed by *Streptococcus pneumoniae* (pneumococcus) (37%) in children and parents (53, 20%). Regardless of HIV status, viruses were detected in higher numbers (44%) in children than their parents (30%) (*p* = 0.049), particularly rhinovirus (*p* = 0.02). Human rhinovirus was the most frequently found virus in both CLH and HUC. Children with adenovirus were at six times increased risk of also having pneumococcus (Odds ratio OR 6, 95% CI 1.12–31.9) regardless of HIV status. In addition, the presence of rhinovirus in children was associated with increased pneumococcal density (Regression coeff 4.5, 1.14–7.9). In CLH the presence of rhinovirus increased the risk of pneumococcal colonization by nearly sixteen times (OR 15.6, 1.66–146.4), and, pneumococcus and *S. aureus* dual colonization by nearly nine times (OR 8.7).

**Conclusions:**

Children more frequently carried viruses regardless of HIV status. In CLH the presence of rhinovirus, the most frequently detected virus, significantly increased co-colonization with pneumococcus and *S. aureus*.

## Introduction

The nasopharyngeal ecosystem composed of respiratory viruses and bacteria is dynamic [1, 2]. Its composition is affected by host, pathogen, and external factors [2, 3]. Underlying HIV infection may affect the density and balance of bacteria and viruses in the nasopharyngeal space.

HIV infected individuals have a high burden of invasive disease from respiratory viruses, and bacteria that may colonize the nasopharynx [4, 5]. HIV increases the risk of pneumococcal and *S. aureus* disease 40–300 times, and nasopharyngeal bacterial colonization usually precedes invasive disease [5, 6]. The association between pneumococcus and *S. aureus* is typically negative in healthy individuals, where if one is present the other usually is not, but in HIV infected individuals, particularly in children living with HIV (CLH), this negative interaction disappears, and CLH are more likely to have dual colonization [7–10].

The carriage density of potentially pathogenic bacteria correlates with pathogenesis, transmission, disease severity, prognosis, and is affected by underlying HIV infection [11–13]. In South Africa, higher carriage density of pneumococcus has been observed in archived nasopharyngeal swabs from CLH compared to HIV uninfected children (HUC) [14]. We also found increased pneumococcal density in CLH compared to HUC [15]. Increased pneumococcal density appears to be an important prognostic marker of pneumococcal pneumonia in adults living with HIV [13].

Respiratory viral infections increase the carriage density of bacteria, specifically pneumococcus, and may impact prognosis in CLH [16]. In hospitalized children with pneumonia in South Africa, 78% had viral infections and CLH specifically, had increased risk of death [17]. Influenza infection in particular was associated with pneumococcal pneumonia in this population.

Access to influenza and pneumococcal conjugate vaccines (PCV) need to reflect these differences in colonization and disease in individuals with HIV, especially in low- and middle-income countries. India has the largest population burden of pediatric HIV outside of sub-Saharan Africa [18], and acute respiratory illness (ARI) is the most common presentation for CLH in India [19]. Vaccine access to PCVs and influenza vaccines are limited for Indian CLH. Understanding nasopharyngeal ecology, the association between viruses with bacteria, and bacterial density in high-risk families could help inform evolving vaccine policy [20–22].

Studies have been focused on the epidemiology of ARI, but not necessarily on the interaction between commensal microbiota and viruses that have the potential to cause severe disease in individuals living with HIV [19, 23]. This study investigates the bacteria and viruses within the nasopharynx, and the association of viruses with bacteria in high-risk families in West Bengal where both children and adults are living with HIV, and access to influenza and pneumococcal vaccines are limited.

## Materials and methods

### Study design

From February 2012 to October 2014, we conducted an interventional prospective cohort study to look at the impact of monovalent *Haemophilus influenzae* type b conjugate vaccine (HibCV) and the 13-valent PCV (PCV13), on nasopharyngeal carriage in children living with and without HIV and their unvaccinated parents, in two rural districts of West Bengal India [24, 25]. During this time, HibCV was introduced in certain states in India but was not part of the program for CLH, and pneumococcal conjugate vaccines were not introduced in India until May 2017. Families living with HIV presenting for routine care were recruited from the antiretroviral treatment center at Midnapore Medical College & Hospital. Families without HIV were recruited from the Hijli Rural Hospital-Kharagpur, a primary health center. None of the study children or their parents had received either HibCV or PCV13 before.

Children living with HIV (*N* = 123; 2–14 years of age) received two doses of catch-up HibCV and one dose of PCV13, and the HIV uninfected children (HUC) (*N* = 44; 2–5 years of age) received a single dose each of HibCV and PCV13 at different time points. To look at the nasopharyngeal carriage, nasopharyngeal (NP) swabs were longitudinally collected from six scheduled visits over a period of 30 months from families living with HIV, and four visits from HIV unaffected families, before and 2 months after each immunization. We banked over 1800 NP swabs which were collected from children and one of their parents at each visit during the entire study. At each visit the child’s illness detail history, antibiotic intake history, vaccination history, ART status, Trimethoprim/Sulfomethoxazole (TMP/SMX) prophylaxis status, latest CD4 count, body weight, height and temperature were noted proximate to swab collection. Nasopharyngeal calcium alginate swabs (Puritan) were collected in 1 mL of skim milk tryptone glucose glycerol (STGG) media, transported at − 4 °C, and banked within 1 h at − 80 °C, at the Indian Institute of Technology (IIT)-Kharagpur.

Hib, pneumococcus, and *S. aureus* were identified by conventional culture methods. HibCV immunization was associated with a significant reduction and a near disappearance in the nasopharyngeal carriage of Hib in CLH and HUC by the last visit [24]. There was no difference in acquisition of vaccine type pneumococcus after one dose of PCV13 in either CLH or HUC [25].

As part of this retrospective nested case-control study, a random selection of 169 archived NP swabs were analyzed by multiplex real time polymerase chain reaction (PCR): 92 swabs from 49 CLH and 43 HUC; and 77 from 44 parents of CLH (PCLH), and 33 parents of HUC (PHUC). These swabs came from the first baseline visit (pre-vaccination; 26 CLH, 23 HUC, 24 PCLH, 15 PHUC) and from the last visit (post HibCV and 2 months post PCV-13 vaccination; 23 CLH, 20 HUC, 20 PCLH, 18 PHUC). Swabs from both the visits were combined and subjected to multiplex real time PCR.

### Multiplex quantitative real time PCR

Bacterial DNA and viral RNA/DNA was extracted from 400 μL of NP swabs with the RTP pathogen kit (Cat No: 1040500200, Stratec®). The genomes were subjected to quantitative multiplex real-time reverse transcription-polymerase chain reaction (rRT-PCR), for detection of respiratory viruses including human adenovirus, human bocavirus, human coronavirus (NL63, 229E, OC43 and HKU1229), enterovirus, influenza A virus, influenza A H1N1 virus, influenza B virus, human metapneumovirus A/B, human parainfluenza virus 1, 2, 3 and 4, human parechovirus, human rhinovirus, RSV A/B, and bacteria including *Streptococcus pneumoniae*, *Staphylococcus aureus*, *Haemophilus influenzae* type b, *Chlamydia pneumoniae,* and *Mycoplasma pneumoniae* as part of the FTD Respiratory pathogens 21 plus kit (Cat No: FTD-2 + .1–64, FTD Diagnostics®). Samples with cycle threshold (Ct) value ≤38 were considered positive. Bacterial loads were calculated using standards provided with the kit, in genomic copies/mL and Log_10_ transformed.

### Statistical analysis

The data was entered into Epi Info 7 (CDC, Atlanta) and statistically analyzed by Stata version 13.1 (STATA Corporation). The bacterial and viral detection rates were defined as the percentage of samples that are positive for a particular bacterium or viral nucleic acid by PCR. Viral subtypes from one group were pooled since rates were low. Categorical variables were analyzed by χ^2^ test, and Wilcoxon rank-sum test was used for continuous. The nutritional status of children was measured by weight-for-age-z scores (WAZ), and height-for-age-z scores (HAZ). Children with Z scores − 2 to − 3 were categorized as moderately malnourished and below − 3 as severely malnourished [26]. CLH were categorized into immunologic categories based on CD4 counts [27]. Risk factor analysis for binary outcomes was done by logistic regression, and by linear regression for ordinal outcomes.

## Results

### Demographic characteristics

The demographics are described in Table 1. The median age of CLH was 4.8 years and 3.5 for HUC. CLH were malnourished and stunted as compared to HUC [WAZ for CLH − 2.456 vs. -1.33 for HUC, *p* = < 0.001]; [HAZ for CLH-1.89 vs. -.92 for HUC, *p* = < 0.001]. 98% of mothers of CLH were living with HIV. Mothers contributed 92% of all parental swabs.

**Table 1:** Demographic characteristics of children and their parents

	Children living with HIV (CLH)	HIV uninfected children (HUC)	***P***
N	49	43	
Age years, median (IQR25, IQR75)	4.827 (3.8, 6.12)	3.53 (3.09, 4.04)	< 0.001*
Female child, n (%)	25 (51)	25 (58)	
WAZ median (IQR25, IQR75)	−2.45 (− 3.63, − 2.043)	−1.33 (− 2.03, −.525)	< 0.001*
HAZ median (IQR25, IQR75)	− 1.89 (− 2.76, − 1.186)	−.925 (− 1.69, .015)	< 0.001*
Child school going, n (%)	27 (55)	28 (65)	0.223
Tuberculosis in house, n (%)	11 (22)	2 (4)	**0.014**
Number of children in house, median (IQR25, IQR75)	2 (2, 2)	2 (1, 2)	0.001*
Number of rooms in house, median (IQR25, IQR75)	2 (2, 3)	2 (2, 4)	0.45
Family income, median (IQR25, IQR75)	11,362 (9478, 11,362)	7594 (7594, 9478)	**0.0004**
*Socioeconomic status*^b^			
Lower, n (%)	0 (0)	2 (4.7)	–
Upper lower, n (%)	2 (4)	10 (23)	**0.006**
Middle, n (%)	20 (41)	29 (67)	**0.01**
Upper middle, n (%)	27 (55)	2 (4.7)	**< 0.00001**
*Fuel used for cooking*			
Gas, n (%)	6 (12)	21 (49)	**.00012**
Wood, n (%)	41 (84)	18 (42)	**.00003**
Coal, n (%)	2 (4)	0 (0)	–
*Parents*			
Mother alive, n (%)	47 (96)	43 (100)	–
Father alive, n (%)	27 (55)	43 (100)	–
Mother HIV infected, n (%)	46 (98)	NA^a^	–
Father HIV infected, n (%)	22 (45)	NA	–
Mothers’ age years, median (IQR25, IQR75)	30 (27, 33)	24 (22, 27)	< 0.001*
Mothers’ schooling years, median (IQR25, IQR75)	7 (3, 9)	9 (7, 10)	0.0004*
Parent on ART during study, n (%)	26 (53)	NA	–

*p < 0.05 significant; ^a^Not applicable, ^b^based on Kuppuswamy’s Socioeconomic Status Scale 2012

### Clinical characteristics

Nearly 36% (18/49) of CLH were on antiretroviral treatment (ART) and 51% were on TMP/SMX prophylaxis (Table 2). 53% of parents of CLH were on ART. The majority of CLH had immune classification of severe [15/49, (30%)] or moderate [22/49, (45%)] disease. The median CD4 count was 650 cells/mm^3^.

**Table 2:** Clinical history of children living with and without HIV

	Children living with HIV (CLH)	HIV uninfected children (HUC)
N	49	43
Immune category, n (%)		
None	12 (24)	–
Moderate	22 (45)	–
Severe	15 (30)	–
Median CD4 count (IQR)	650 (392, 912)	–
On ART, n (%)	18 (36)	–
On TMP/SMX, n (%)	25 (51)	–
Median ART Duration (IQR), month	3 (2, 5)	–
HIV plasma load, median IQR	2.57E4 (3.54E3, 1.36E5)	–
Tuberculosis co-morbidity, n (%)	5 (10)	0
Ear infection history, n (%)	6/26 (23)	1/23 (4)
Past week sickness history, n (%)	14 (28)	10 (23)
Fever, n (%)	7 (14)	5 (11)
Cough, n (%)	7 (14)	6 (14)
Rhinorrhea, n (%)	2 (4)	5 (11)
Diarrhea, n (%)	1 (2)	0
Antibiotic intake during past week, n (%)	7 (14)	4 (9.3)

We noted the clinical history of children prior to NP specimen collection, and found 28% of CLH, and 21% of HUC, were sick in the past week. The major symptoms included fever, cough, rhinitis or diarrhea; however, during swab collection all children were afebrile (< 100.4 °F) and asymptomatic. Over 23% of CLH had a history of ear infection within 1 month prior to study, compared to 4% in HUC (*p* = 0.06), suggesting the increased risk of otitis media in CLH.

### Nasopharyngeal bacteria and viruses in children versus parents

A total of 169 NP swabs (92 from children; 77 from parents) were tested for viruses and 147 (83 from children, 64 from parents) for bacteria (Table 3). Bacteria were identified in 67% of the children, and 54% of parents. *Staphylococcus aureus* was the most common bacterium identified in children [53%, 44/83], followed by *Streptococcus pneumoniae* [37.3%, 31/83], and *Chlamydia pneumoniae* [2%, 2/83]. Similar trends were seen in their parents, [*S. aureus* (53%); pneumococcus (20%)]. Higher rates of pneumococcus were found in children compared to parents (*p* = 0.02).

**Table 3:** Nasopharyngeal bacteria and viruses identified in children and their parents

	Children	Parents	***P*** value
	No. (%)	No. (%)	
**Bacterial detection**			
N	83	64	
No. of individuals with no bacteria^a^	26 (31)	26 (40.6)	0.24
No. of individuals with bacteria^b^	56 (67)	35 (54.6)	0.11
No. of individuals with bacteria only^c^	34 (41)	27 (42)	0.88
Total *S. pneumoniae*^d^	31 (37)	13 (20)	**0.02**
Total *S. aureus*	44 (53)	34 (53)	0.98
Total *C. pneumoniae*	2 (2.4)	0	–
> 1 bacteria in one swab^e^	24 (29)	11 (17)	0.098
*S. pneumoniae + S. aureus*	20 (24)	11 (17)	0.30
*S. pneumoniae + C. pneumoniae*	1 (1)	0	–
**Virus detection**			
N	92	77	
Number of individuals with virus^f^	41 (44.5)	23 (30)	**0.049**
Single virus only^g^	3 (3)	9 (11.7)	0.03
Human rhinovirus	15 (16)	4 (5)	**0.02**
Human adenovirus	9 (10)	6 (8)	0.65
Human bocavirus	6 (6)	0	–
Influenza B virus	4 (4)	0	–
Respiratory Syncytial Virus	2 (2)	5 (6.5)	0.16
Human coronavirus	2 (2)	1 (1)	0.66
Human metapneumovirus	2 (2)	0	–
Human parainfluenza virus	1 (1)	2 (2.6)	0.45
Human enterovirus	0	3 (4)	–
**Viruses + bacteria co-detection**			
N	83	64	
Total viruses + bacteria^h^	22 (26.5)	9 (4)	0.06
Single virus + bacteria	12 (14.4)	8 (12.5)	0.73
Mixed virus + bacteria	10 (12)	1 (1.5)	**0.01**

^a^Individuals not having any bacteria; ^b^Number of individuals with bacteria detection singly or with other bacteria with or without virus; ^c^Number of individuals with bacteria detection singly or with other bacteria without virus; ^d^Total bacteria detected individually or in combination with other bacteria or viruses in individuals; ^e^Mixed bacteria detected with other bacteria; ^f^Total virus detected individually or in combination with other viruses or bacteria; ^g^One virus detected in an individual without bacteria; ^h^Total viruses detected with bacteria

Pneumococcus with *S. aureus* were the most frequent bacteria co-detected (24%) in children. Viruses were detected in higher numbers (44%) in children than their parents (30%) (*p* = 0.049), particularly rhinovirus (*p* = 0.02). Viruses with bacteria were more frequently co-detected in children (26%), than virus alone (3%). For example, 13 out of 15 times rhinovirus was detected with bacteria, and adenovirus was found with bacteria 8 out of 9 times.

### Nasopharyngeal microbes and viruses in children with and without HIV

#### Bacteria detection

Bacterial pathogens were tested in specimens from 43 CLH, 40 HUC, and viruses in 49 CLH, 43 HUC (Table 4). Samples for bacterial testing were lower due to the loss of reactions in bacterial standards.

**Table 4:** Nasopharyngeal bacteria and viruses identified in children living with and without HIV

	Children living with HIV	HIV uninfected children	P
	n, (%)	n, (%)	
**Bacteria**			
n	43	40	
No. of individuals with bacteria only^a^	18 (42)	16 (40)	0.86
No. of individuals with bacteria^b^	30 (70)	26 (65)	0.64
Total *S. pneumoniae*^c^	16 (37)	15 (37)	0.98
Total *S. aureus*^c^	27 (63)	17 (42)	0.06
Total *C. pneumoniae*^c^	1 (2)	1 (2)	0.96
> 1 bacteria in one swab	14 (32)	7 (17)	0.11
*S. pneumoniae + S. aureus*	13 (30)	7 (17)	0.17
*S. pneumoniae + C. pneumoniae*	1 (2)	0	–
**Viruses**			
N	49	43	
No. of individuals with virus^d^	20 (41)	21 (49)	0.43
Human rhinovirus	7 (14)	8 (18)	0.57
Human adenovirus	7 (14)	2 (4)	0.12
Human bocavirus	2 (4)	4 (9)	0.31
RSV	1 (2)	1 (2)	0.92
Human coronavirus	1 (2)	1 (2)	0.92
Human metapneumovirus	1 (2)	1 (2)	0.92
Human parainfluenzavirus	1 (2)	–	–
Influenza b virus	–	4 (9)	–
Human enterovirus	–	–	–
**Viruses + Bacteria**			
N	43	40	
Total viruses + bacteria	12 (28)	10 (25)	0.76

^a^ Number of individuals with bacteria detection singly or with other bacteria without virus; ^b^Number of individuals with bacteria detection singly or with other bacteria with or without virus; ^c^Total number of bacteria detected individually or in combination with other bacteria in individuals;^d^Number of individuals with viruses detected individually or in combination with other viruses or bacteria

Bacteria were identified in 70% of CLH and 65% of HUC. *S. aureus* was identified in an increasing fashion in CLH, as compared to HUC (63% vs. 42%; *p* = 0.06). Similar pneumococcus and *C. pneumoniae* rates were found in CLH and HUC (37%; 2% each). Nearly 69% (11/16) of CLH with pneumococcus and 51% (14/27) of CLH with *S. aureus* were on TMP/SMX prophylaxis. This suggests TMP/SMX did not decrease their risk for carriage. Co-occurrence of pneumococcus with *S. aureus* was found more often in CLH (30%), than in HUC (17%), although not significant (*p* = 0.17). We could not detect Hib by PCR in both the groups regardless of HibCV vaccination status.

#### Respiratory virus detection

In the 92 samples tested, viruses were detected in 41% of CLH and 49% of HUC. Rhinovirus and adenovirus were most frequently detected in CLH (14% each), followed by bocavirus 4%, RSV 2%, coronavirus-229 2%, human metapneumovirus 2% and human parainfluenza-3 virus (2%). Of interest, viruses were identified mainly in children with stage 2 and 3 HIV disease (13/14), than stage 1 (1/14) (*p* = < 0.001). 57% of CLH with viral positivity were not on ART. Rhinovirus was also most frequent in HUC (18%). Influenza B was identified only in HUC (9%). Being on an antibiotic in the past week, increased the risk of virus detection by 3.85 times, (95% CI 1.05–14, *p* = 0.04).

#### Virus-bacteria co-detection

The co-occurrence of ≥1 bacteria with ≥1 viruses was found in 28% of CLH, and 25% of HUC. The co-occurrence was mainly found in children with stage 2 and stage 3 HIV disease (11/12), as compared to stage 1 (1/12) (*p* < 0.001). Interestingly, wood used for cooking within households, increased the risk of virus (OR 3.09, 1.0–9.21, *p* = 0.042) and virus-bacteria detection (OR 4.75, 1.2–17, *p* = 0.019) among children, suggesting indoor air pollution may increase the risk for viral circulation.

### Nasopharyngeal bacteria and viruses identified in parents with and without HIV

Bacteria were tested in 33 PCLH and 31 PHUC specimens, and viruses in 44 PCLH and 33 PHUC. Bacteria were detected in 51% of PCLH and 58% of PHUC. *S. aureus* was most frequent in both PCLH (51%) and PHUC (58%). Similar rates of pneumococcus (20–21%) were detected in both parents, and their children. Viruses were detected in 34% of PCLH and 27% of PHUC. Adenovirus was most common in PCLH (11%), and RSV in PHUC (12%). Virus-bacteria co-detection was found in 16% of PHUC and 12% of PCLH.

### Co-detection of nasopharyngeal bacteria with viruses

Virus-bacteria co-detections were found in 12 CLH and 10 HUC. Of the 12 CLH, 7 had co-occurrence of *S. pneumoniae* + *S. aureus* with viruses. The remaining 5 CLH had co-occurrence of viruses with pneumococcus (2), or *S. aureus* (3). Among the HUC, viruses were co-detected with pneumococcus (3), *S. aureus* (3), *S. pneumoniae* + *S. aureus* (3), or, *C. pneumoniae* (1). Among parents, viruses were co-detected only with *S. aureus* (8/64).

### Nasopharyngeal carriage in children with and without history of symptoms

A history of respiratory symptoms in the past week was associated with more than 4 times increased risk of virus detection in children (OR 4.2; 1.5–11.7; *p* = 0.005), and 3.65 times virus-bacteria co-detection (OR 3.65; 1.3–10; *p* = 0.01). CLH with symptoms in the past week, had 6 times increased risk of virus detection (6.44; 1.62–25; *p* = 0.008), as compared to 2 fold in HUC (2.6; .56–12; *p* = 0.22).

### Association of viruses with *S. pneumoniae* and its nasopharyngeal density in children

*S. pneumoniae* was associated with co-occurrence of viral species in children regardless of their HIV status (OR 3.9) (Table 5), particularly adenovirus co-detection increased the likelihood of pneumococcal detection six fold (*p* = 0.036). Increased pneumococcal density was seen with co-detection of viruses (Coeff 3.6), multiple viruses (Coeff 5), and rhinovirus specifically (Coeff 4.5) in children regardless of their HIV status.

**Table 5:** Association between isolation of *Streptococcus pneumoniae* with respiratory viruses, and, between the density (Log copies/ml) of *Streptococcus pneumoniae* with viruses in the nasopharynx of children

	Pneumococcal colonization				Pneumococcal density			
	n^a^	OR^b^	95% CI^c^	P	n	Coeff^d^	95% CI	P
**Overall children**								
Virus presence	83	3.93	1.47–10.5	0.006	31	3.603	.44–6.7	0.027
1 virus	83	3.85	1.15–12.8	0.028	31	0.533	−3.2-4.3	0.77
> 1 virus	83	2.25	0.62–8.1	0.21	31	5.06	1.1–9	0.01
Rhinovirus	83	3.13	0.99–9.9	0.052	31	4.52	1.1–7.9	0.01
Adenovirus	83	6	1.12–32	0.036	31	1.62	−2.7-6	0.45
**Children living with HIV**								
Rhinovirus	43	15.6	1.66–146	0.016	16	4.22	−0.2-8.6	0.06
Rhinovirus + *S. aureus*	43	8.7	1.42–54	0.019				
Adenovirus	43	4.16	0.66–26	0.12	16	−0.46	−6.1, 5.1	0.86
**HIV uninfected children**								
Rhinovirus	40	1	.20–4.9	1	15	2.7	−1.9-7.3	0.23
Adenovirus	40	NA^e^	NA	NA	15	2.77	−2.7, 8.3	0.3

^a^*n* number of observations; ^b^*OR* Odds ratio; ^c^*CI* Confidence interval; ^d^*Regression coefficient;* ^e^*NA* Not available

In CLH, the presence of rhinovirus increased the risk of pneumococcal colonization by nearly sixteen times (OR 15.6), and pneumococcus and *S. aureus* dual colonization increased nearly 9 times in the presence of rhinovirus (OR 8.7).

## Discussion

This is the first report from India about household level prevalence and association of respiratory viruses with bacteria in parents and children in the context of HIV, using multiplex real time PCR. In this study, we found higher prevalence of bacteria and viruses in children than their parents regardless of HIV status. This suggests parents or caregivers are at increased risk of exposure to viruses and bacteria in households with children. This is in agreement with the results of our immunization study where we found bacterial carriage within the households-- carriage in children predicted the carriage in parents and thus immunization in children indirectly impacts the carriage in unvaccinated adults.

A majority of the CLH had moderate to severe HIV disease and were not on ART, which increased their risk for bacteria and virus detection [28]. CLH living in households with adults with HIV who are also at high risk, puts these families at increased risk for severe invasive diseases. Similar to our findings, others have reported more *S. aureus* carriage in CLH, as compared to HUC [29–31], while some did not [10]. Although the swabs in this study came from an unimmunized and HibCV and PCV13-immunized cohort of children, the overall pneumococcal carriage was not affected. Rather, we detected higher rates of pneumococcus (37%) by PCR than by culture detected pre (31%) and post PCV13 (28%) immunization [25, 32]. Further, we observed in the past that the point prevalence of pneumococcus did not change with one dose of PCV13 in both CLH and HUC [25]. The differences were noted only in serotypes and the density of carriage [25, 33]. The overall rates of pneumococcus in CLH were similar to studies from Brazil (28%) [34], and Indonesia (46%) [35]. While reasons are not clear, similar pneumococcal detection rates have been seen between CLH and HUC by us in this cohort [25], in Brazil [34], Mozambique [36], and South Africa [7]. Serotype specific carriage may provide more information; as seen earlier, HIV infection increased the risk of colonization of non-vaccine type and antibiotic resistant pneumococcal serotypes [32]. TMP/SMX prophylaxis did not appear to influence the carriage of pneumococcus or *S. aureus*. This could be due to the resistance of pneumococcal isolates to TMP/SMX, as we saw in 98% of isolates in the past [32].

The co-colonization of otherwise competing pneumococcus and *S. aureus* in those living with HIV could be immune-mediated due to decrease in pneumococcal-specific CD4^+^T cells in HIV infected individuals [37]. The interference, or negative association, between pneumococcus and *S. aureus* seen in HIV uninfected children, has been observed following antiretroviral treatment in CLH [7].

Rhinovirus and pneumococcus have been observed to be positively associated in healthy children under-2 [2, 38]. An Indian study reported rhinovirus with *S. aureus* in symptomatic individuals living with HIV [39]. We also found a positive association of rhinovirus, *S. aureus,* and pneumococcus in CLH, when they were asymptomatic.

Viral infections commonly cause hospitalizations in CLH, and rhinovirus and adenovirus in particular are responsible for LRTI and acute otitis media (AOM) in both HUC and CLH [3, 17, 40]. Higher rates of viruses were detected in children regardless of HIV, particularly rhinovirus. Rhinovirus has been the most frequently identified virus in asymptomatic children, also found in a healthy control group of a childhood pneumonia study in Asia, Africa and South America [2, 41, 42]. The detection of virus in the nasopharynx may suggest asymptomatic carriage, subclinical infection, or prolonged viral shedding from recent symptomatic infection, or a past infection [43, 44]. CLH may shed viruses for a longer time, and this may increase their risk for secondary bacterial infections leading to pneumonia and AOM, and also increase risk of infections within families where both adults and children are living with HIV [20, 45, 46].

Otitis media and LRTI cause significant morbidity and mortality in CLH [47]. Bacterial and viral interactions specifically involving rhinovirus, adenovirus, pneumococcus, *S. aureus*, and nontypeable *H. influenzae* are particularly important in the pathogenesis of these conditions [3]. In studies in South Africa with HUC, CLH, and HIV exposed uninfected children, CLH had the same spectrum of pathogens for these conditions; however, they had increased risk of mortality [17].

Association of increased pneumococcal density with viruses, has been observed during asymptomatic [48], and symptomatic viral infections [16, 21, 49]. Respiratory viral co-infections are demonstrated to be associated with invasive pneumococcal pneumonia [21]. Viral co-detection with increasing pneumococcal load without producing symptoms, may suggest that viruses promote growth and transmission of bacterial colonizers in the nasopharynx, thus predisposing individuals to complications of upper respiratory tract infections [48]. Rhinovirus co-infections were associated with increasing pneumococcal density during episodes of ARI in young children in rural Peru [50]. A Finnish study showed rhinovirus circulation was associated with invasive pneumococcal disease (IPD) in children [45]. Mechanistic in vitro studies support that rhinovirus increases pneumococcal adherence, through the expression of platelet-activating factor receptor [51], and disrupts the epithelial barrier functions thus promoting the binding, translocation, and persistence of bacteria [52]. Similarly, adenovirus has been correlated with IPD in children [21, 53] and in vitro [54].

The major findings of this study are the detection of viruses with increasing pneumococcal density in asymptomatic children, and rhinovirus co-detection with pneumococcus in CLH compared to age-matched HUC. Association between pneumococcus, *S. aureus,* and rhinovirus in CLH, supports the increased risk of polymicrobial pneumonias in CLH. Pneumococcal conjugate vaccines impact the nasopharyngeal ecology and the risk of LRTI and AOM in adults and children with HIV [7, 55, 56].

The study is limited in its sample size, therefore confidence intervals were wide for OR, and we could not do multivariate analysis for association between microbes. The swabs were part of a vaccine impact study and were combined from pre and post immunization visits, so there was an inherent bias in the selection; however, the overall pneumococcal carriage was not affected. The clinical relevance of viral activity with increasing pneumococcal density in asymptomatic children requires further study through well designed cohort studies.

Pneumococcal-viral interactions are complex. Larger longitudinal studies are needed to understand the viral-bacterial dynamics in high-risk families, in both asymptomatic, and symptomatic situations. Viral load, and host immune responses, may better differentiate asymptomatic versus active viral infections [57]. India has a huge burden of HIV and pneumonia. Pneumococcal vaccines are being rolled-out in the Indian UIP but seasonal influenza vaccines are still not part of government programs and neither are available in programs for individuals living with HIV. It is essential to think about access to vaccines for children and adults with HIV to mitigate respiratory infections [58]. This study is timely in looking at the association of pneumococcus with viruses in this high-risk group.

In conclusion, this study demonstrates high detection of viruses and bacteria in asymptomatic children with and without HIV and shows that pneumococcal density increases in the presence of viruses.

## Data Availability

The datasets during and/or analyzed during the current study available from the corresponding author on reasonable request.
